# Ovarian tissue cryopreservation for fertility preservation: clinical and research perspectives

**DOI:** 10.1093/hropen/hox001

**Published:** 2017-03-29

**Authors:** Richard A. Anderson, W. Hamish B. Wallace, Evelyn E. Telfer

**Affiliations:** 1 Medical Research Council, Centre for Reproductive Health, Queen's Medical Research Institute, 47 Little France Crescent, EdinburghEH16 4TJ, UK; 2 Department of Haematology/Oncology, Royal Hospital for Sick Children, 9 Sciennes Rd, Edinburgh EH9 1LF, UK; 3 Institute of Cell Biology and Centre for Integrative Physiology, University of Edinburgh, Hugh Robson Building, George Square, Edinburgh EH8 9XD, UK

**Keywords:** cryopreservation, gonadotoxicity, fertility preservation, tissue culture, ivm: artificial ovary

## Abstract

**BACKGROUND:**

Small case series have reported successful live births after ovarian tissue cryopreservation and orthotopic transplantation, demonstrating that it can be of value in increasing the chance of successful pregnancy after treatment for cancer and other fertility-impacting diseases in adult women.

**OBJECTIVE AND RATIONALE:**

This review is intended to set out the current clinical issues in the field of ovarian tissue cryopreservation, and elucidate the status of laboratory studies to address these.

**SEARCH METHODS:**

We reviewed the English-language literature on ovarian tissue cryopreservation and in vitro maturation (IVM) of ovarian follicles.

**OUTCOMES:**

Ovarian tissue cryopreservation is increasingly used for fertility preservation and, whilst areas for development remain (optimal patient selection, minimizing risk of contamination by malignant cells and IVM protocols), there are emerging data as to its efficacy. We review the current status of ovarian tissue cryopreservation in girls and young women facing loss of fertility from treatment of cancer and other serious diseases. Increasingly large cohort studies are reporting on success rates from ovarian tissue cryopreservation giving an indication of likely success rates. Patient selection is necessary to ensure the safety and effectiveness of this approach, especially in the very experimental situation of its application to prepubertal girls. There are continuing developments in supporting follicle development *in vitro*.

**LIMITATIONS, REASONS FOR CAUTION:**

The evidence base consists largely of case series and cohort studies, thus there is the possibility of bias in key outcomes. *In vitro* development of human ovarian follicles remains some way from clinical application.

**WIDER IMPLICATIONS OF THE FINDINGS:**

Ovarian tissue cryopreservation is becoming established as a valuable approach to the preservation of fertility in women. Its application in prepubertal girls may be of particular value, as it offers the only approach in this patient group. For both girls and young women, more accurate data are needed on the likelihood of successful childbirth after this procedure and the factors that underpin successful application of this approach, which will lead to its more effective use.

**STUDY FUNDING/COMPETING INTERESTS:**

The author's work in this field is supported by Medical Research Grant (MRC) grants G0901839 and MR/L00299X/1 and partially undertaken in the MRC Centre for Reproductive Health which is funded by MRC Centre grant MR/N022556/1. The authors declare that there is no conflict of interest that could prejudice the impartiality of the present research.

**WHAT DOES THIS MEAN FOR PATIENTS?**

Freezing part of an ovary before treatment for cancer and other diseases that might lower fertility can provide women with a better chance of having a baby once they have recovered. Approximately 100 successful live births have occurred after replacing this ovarian tissue.

The procedure of freezing ovarian tissue (cryopreservation) and later transplanting it back to the body raises a number of important clinical questions. This review identified what these main issues are and what studies have been, and still need to be, carried out.

Increasing the use of ovarian cryopreservation is providing results on the likely successful birth rates but more information is required. It may be more suitable for some women than others and careful patient selection will be needed, especially to get a more accurate prediction of the risk of loss of fertility to the individual patient. Researchers are also working on better ways to support growth of follicles (where immature eggs, or oocytes, are found) in the laboratory, for future use in the IVF clinic.

Studies show that freezing of ovarian tissue for later transplantation is a major advance for fertility preservation. However, it is not suitable for all patients as there may be concerns about transplanting cancer cells into disease-free patients. The key areas for further study are optimal patient selection, minimizing risk of contamination by cancer cells and improving laboratory protocols for oocyte maturation.

## Introduction

Advances in treatment of many cancers with consequent dramatic improvements in long-term survival (Miller *et al*., 2016) have led to increasing awareness of the issues in what is sometimes termed ‘survivorship’ to encompass the wide range of medical and social issues faced by cancer survivors. Amongst medical issues, the potential effects on fertility are an important concern of young women facing therapy for cancer and other conditions treated with similar chemotherapeutic regimens (Peate *et al*., 2011), and the field of fertility preservation has grown rapidly over the last two decades (Ataman *et al*., 2016). The most common cancers in girls and young women are those of the breast, cervix, central nervous system, leukaemias and lymphomas. Treatment of all these conditions may compromise fertility in girls and young women as a result of the surgery, chemotherapy and radiotherapy required for treatment.

**Figure 1:**
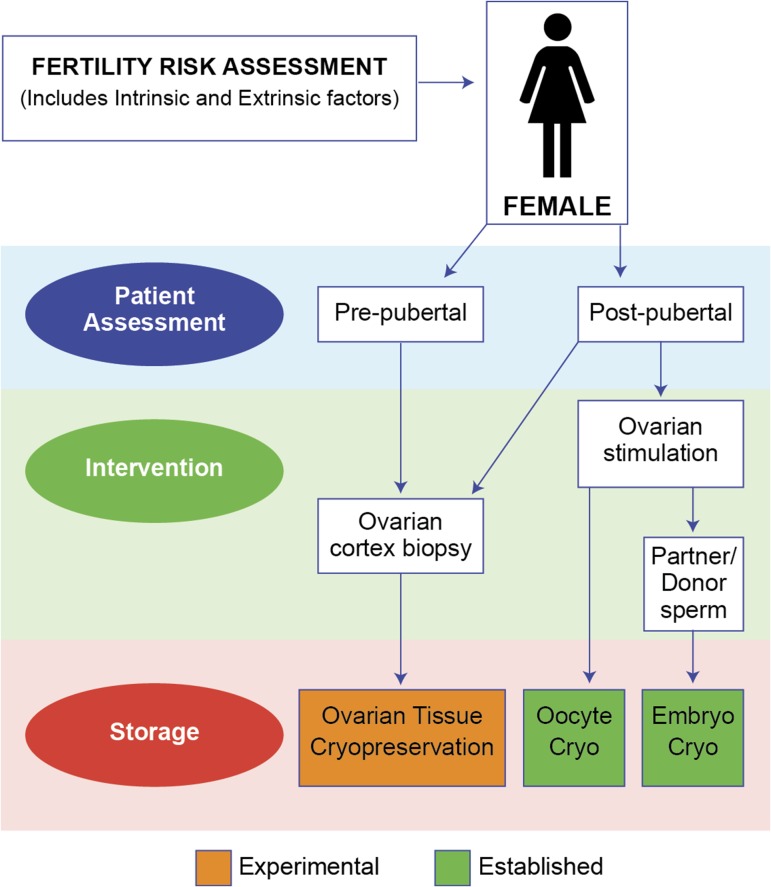
Ovarian tissue cryopreservation within the wider context of fertility preservation for girls and women. Cryo: cryopreservation. Adapted from Anderson *et al*. (2015).

Ovarian tissue cryopreservation is one of several options available for female fertility preservation, together with oocyte and embryo cryopreservation (Loren *et al*., 2013; Anderson *et al*., 2015) (Fig. 1). Other approaches include administration of agents that might protect the ovary, such as GnRH analogues (Lambertini *et al*., 2015), and surgical transposition of the ovary to reduce the radiation dose (Gubbala *et al*., 2014). This review will focus on ovarian tissue cryopreservation and address some of the outstanding issues involved in the development of this approach, including issues surrounding patient selection, the evidence for its efficacy and the current status of attempts to circumvent one of the significant issues with this technique, i.e. the potential presence of malignant cells contaminating the cryopreserved tissue.

The potential value of cryopreservation of ovarian tissue was first demonstrated in a series of experiments in the sheep where autografts of cryopreserved ovarian cortex were sutured on to the ovarian pedicle, with subsequent restoration of ovarian cyclicity and natural conception (Baird *et al*., 1999). These key experiments demonstrated the potential of this approach for clinical application but the detailed histological analysis that accompanied them also showed one of the major issues, which is that the majority of primordial follicles in the cryopreserved tissues do not survive. This is thought to be due to ischaemia prior to revascularization of the graft rather than direct cryo-damage and there is, as yet, no clear way of markedly improving this. The advent of vitrification, which has transformed the cryopreservation of mature oocytes, may offer some advantages in reducing the cryo-damage that does occur (Ting *et al*., 2011; Amorim *et al*., 2012; Zhou *et al*., 2016), and indeed has been successfully used clinically in this context, but it does not address the problem of revascularization. One approach to that problem is the cryopreservation of the whole ovary with subsequent vascular re-anastomosis, and whilst there have been developments towards successful use of this approach in animal models (Campbell *et al*., 2014), it remains some way from clinical practice.

## Search Methods

We reviewed the English-language literature on ovarian tissue cryopreservation and IVM of ovarian follicles using conventional search methods. This was supplemented with searching cited publications in identified reports, and personal contacts with several key researchers.

## When and for whom is ovarian tissue cryopreservation appropriate?

This question raises much broader issues than strictly medical ones, and includes assessment of risk of loss of fertility, patient autonomy, decision-making at a time of stress and potential information overload, and cost (either to the patient or the health service). This can, in part, be perceived as the need to strike a balance between offering fertility preservation to all at risk, or only to those with a clear need, i.e. at high risk. Part of the evidence base relevant to this is analysis of which cancer treatments clearly do or do not impact on female fertility. The most detailed information on this is derived from the US Childhood Cancer Survivor Study (CCSS) which has for many years published analyses of reproductive outcomes in a cohort of cancer survivors using their siblings as the control group (Chemaitilly *et al*., 2006; Signorello *et al*., 2006; Green *et al*., 2009). A recent report from this study analysed chemotherapy exposures in relation to the prevalence of pregnancy and live birth in women who had been exposed to chemotherapy: females who had received cranial or pelvic radiotherapy were excluded from this analysis (Chow *et al*., 2016). The overall hazard ratio for pregnancy was 0.87 (95% CI: 0.81–0.94) compared with the control group of their siblings, demonstrating that most women who have had chemotherapy for cancer treatment in childhood will be able to have children. Detailed analysis of the particular agents involved identified that alkylating agents were only associated with a decrease in the risk of pregnancy and live birth at the highest doses. A similar result was reported from a recent analysis of survivors of Hodgkin lymphoma in childhood and adolescence from Germany, in which the overall chance of parenthood of Hodgkin lymphoma survivors was very similar to that of the German population (Bramswig *et al*., 2015). They showed that procarbazine in cumulative doses up to 11 400 mg/m^2^ and cyclophosphamide in cumulative doses up to 6000 mg/m^2^ had no significant or only minor effects on parenthood. The chance of parenthood was however significantly reduced in survivors receiving pelvic radiation. These studies also highlight that it is the treatment rather than the diagnosis that determines the risk, and also that the majority of young cancer survivors will go on to have the opportunity to have a family.

This information underpins the other aspect of the argument, whether this approach should be primarily aimed at those with a clear need, i.e. where a significant risk of loss of fertility is predicted based on the planned treatment. Ovarian tissue cryopreservation for restoration of fertility remains widely regarded as an experimental procedure (Loren *et al*., 2013), although increasing experience with it has led to it becoming established practise in Israel (Meirow *et al*., 2016). In our view, patients having ovarian tissue cryopreservation should be counselled and have their data collected in a research context, to improve the evidence base underpinning this approach. This is particularly important for children and younger adolescents where proxy consent from parents and carers is often the norm (Wallace *et al*., 2016). It remains important to avoid unnecessary intervention or procedures of uncertain benefit in unwell children at a time of extreme stress to the patient and their parents and carers. To provide a structure for discussions surrounding whether or not to proceed with ovarian tissue cryopreservation, we have developed a risk assessment tool, dividing the key issues into those intrinsic to the patient, and those extrinsic (Table I)(Wallace *et al*., 2012).

**Table I:** Intrinsic and extrinsic factors that should be taken into account when considering fertility preservation strategies for girls and young women undergoing treatment.

Intrinsic factors
Health status of the patient
Psycho-social factors
Consent (patient/parent)
Assessment of pubertal status
Assessment of ovarian reserve
Extrinsic factors
Nature of predicted treatment (high/medium/low/uncertain risk)
Time available
Expertise/technical options available

Reprinted with permissions from Anderson *et al*. (2015).

The intrinsic factors include whether the patient is well enough to have the necessary laparoscopic surgery to remove ovarian tissue. This should include assessment of coagulation disorders, infection risk, issues regarding the risk of anaesthesia and the potential risk of contamination of ovarian tissue by malignant disease (the latter is discussed more fully below). Issues regarding informed consent have been mentioned above. Whilst there is no theoretical lower age limit for the potential value of ovarian tissue cryopreservation, the decline in the ovarian reserve (Wallace and Kelsey, 2010), and in natural fertility in the later 30s and the consequent chances of success of the procedure in terms of restoring fertility should be clearly discussed. Assessment of the ovarian reserve using biomarkers such as anti-Müllerian hormone (AMH) or antral follicle count are routinely performed prior to assisted reproduction to predict the patient's response (Iliodromiti *et al*., 2014). Many studies have shown a reduction in these biomarkers after cancer treatments indicating their potential to quantify ovarian toxicity and distinguish between the effects of different regimens (Bath *et al*., 2003; Lutchman Singh *et al*., 2007; Lie Fong *et al*., 2008; Decanter *et al*., 2010). Pretreatment levels of AMH have also been shown to predict the risk of amenorrhoea in women subsequently treated with chemotherapy for breast cancer (Anderson and Cameron, 2011; Su *et al*., 2014), substantiating the hypothesis that women with a higher ovarian reserve pretreatment are more likely to have ongoing ovarian function thereafter. Such studies have not been performed with fertility as an outcome but this approach does seem to have some promise to individualize more accurately the risk of specific therapies. It would seem likely that these biomarkers could be used to give some prediction of remaining postchemotherapy ovarian function and reproductive lifespan. Similar analyses have been performed in healthy women, but accurate prediction of remaining reproductive lifespan seems only poorly predicted by, for example, AMH, except at particularly low levels in younger women (van Disseldorp *et al*., 2008; Tehrani *et al*., 2013). Even then, sporadic ovulation in young women will allow some to conceive. The limited value of these markers for prediction reflects our poor understanding of ovarian compensation after such damage, and this is an area where research could have a marked impact on clinical practice.

The key extrinsic factor is the nature of the predicted treatment and, as mentioned above, the key risk factors are the administration of whole body, pelvic (or abdominopelvic in children) radiotherapy and high-dose alkylating agents. The patient's age is probably the most important determinant of how damaging these therapies are, as reflected in the dramatic increase in risk of amenorrhoea after chemotherapy in women treated for breast cancer with older age at treatment (Petrek *et al*., 2006). However, a significant problem is that even therapies that have low risk, such as the commonly administered ABVD regimen for early stage Hodgkin lymphoma, may have some impact on ovarian function (potentially in those with lower pretreatment ovarian reserve) as women treated with this regimen have recently been shown to have a reduced oocyte yield when oocyte collection for cryopreservation is subsequently performed following relapse of the disease (Sonigo *et al*., 2016), although no effect on risk of early menopause (before the age of 40 years) has been reported (Swerdlow *et al*., 2014). This also highlights that whereas initial therapy for the primary malignancy may carry a low risk of premature ovarian insufficiency (POI) and infertility, treatment may change depending on initial response (e.g. in lymphoma: Barrington *et al*., 2016) and regimens used for relapse will generally carry a high risk. Ovarian tissue cryopreservation and restoration of fertility potential is likely to be more effective if those patients had undergone fertility preservation procedures at the time of initial diagnosis. Treatment protocols to minimize the risk of recurrence in combination with reducing general, including gonadal, toxicity are continually evolving.

The potential value of careful prediction of risk of treatment to ovarian function has been supported by an analysis of ovarian function in a population of children and young adults aged up to 18 years, some of whom were offered ovarian tissue cryopreservation (Wallace *et al*., 2014). This showed that those who met the criteria and were therefore offered ovarian tissue cryopreservation had a substantial risk of POI during the course of the period of follow-up, of 35% over the 15 year follow-up period. This contrasted with a prevalence of only 1% (1 individual in a cohort of 141) in those not offered ovarian tissue cryopreservation giving a hazard ratio of 56.8 (95% CI: 6.2–521.6) at 10 years follow-up. The young age of the population studied precluded the use of fertility as the key outcome but this clearly shows the potential for focussed patient selection. In contrast, in a relatively unselected population of women who underwent pre-chemotherapy unilateral oophorectomy for fertility preservation, follow-up at a mean of 58 months showed a prevalence of POI of 9% in women with breast cancer, and 18% in those with lymphoma, but 87% of those with leukaemia, reflecting the widespread use of bone marrow transplantation in that group (Schmidt *et al*., 2013). Overall, 41 of the 57 women in the group who had tried to conceive had been successful, i.e. a retained fertility rate of 72%. Whilst the issues surrounding the provision of all aspects of fertility preservation require a much more complete evidence base than is currently available, it seems clear that one aspect that should be addressed is the need for more accurate identification of those who are very likely to retain their fertility after their cancer treatment, which will guide both patients and medical services in the provision of this treatment option.

## How effective is ovarian tissue cryopreservation?

The first successful pregnancy after replacement of cryopreserved ovarian tissue was reported in 2004 (Donnez *et al*., 2004) and at present there are ~100 babies born following this procedure, although this number is increasing all the time. An analysis of 60 tissue replacements carried out in Belgium, Denmark and Spain demonstrated that over 90% of women showed some evidence of ovarian activity, first seen a median of 4 months after transplantation (Donnez *et al*., 2013). Eighteen per cent of these women achieved a pregnancy, the majority after natural conception, and 12 live births were recorded from 6 women. An updated analysis including additional centres (total 111 women) indicated a 29% pregnancy rate. An analysis of 41 transplantations in Denmark showed that 31% of those wishing to conceive had a successful pregnancy (Jensen *et al*., 2015): 100 replacements have now been performed there (C.Y. Andersen, personal report). A recent report has presented data on 95 orthotopic cryopreserved ovarian tissue transplantations in 74 women in the FertiProtekt Network (Van der Ven *et al*., 2016). These women had an average age of 30 years at cryopreservation and 34 years at transplantation, with the two most common diagnoses being breast cancer and Hodgkin lymphoma. Amongst those undergoing their first transplantation who had clear evidence of POI at the time of transplantation, 62.5% showed evidence of ovarian activity 1 year after transplantation, and 27.5% achieved a pregnancy with delivery in 22.5%. These case series provide, at present, the best estimate of the likely success of this approach, although no doubt additional pregnancies will be achieved, increasing the overall success rate. Indeed, there are case reports of individual women having up to three successful pregnancies after ovarian tissue replacement, with graft function lasting up to 10 years (Jensen *et al*., 2015). Calculation of success rate is however complicated by a range of issues including that some transplantations are not being performed to restore fertility but for endocrine reasons, and that some women have some residual ovarian activity at the time of transplantation (Andersen, 2015).

The majority of these pregnancies were after natural conception, and the potential for this is one of the key positive aspects of ovarian tissue cryopreservation compared with oocyte or embryo cryopreservation. A more aggressive approach with repeated cycles of ovarian stimulation to either achieve pregnancy with fresh embryo replacement (Meirow *et al*., 2016) or, indeed, to bank cryopreserved embryos whilst the ovarian graft remains functional (Oktay *et al*., 2016) has also been reported. In one series of 20 women, some of whom had previously received chemotherapy for leukaemia or lymphoma, 32% had a baby after up to 11 cycles of IVF (Meirow *et al*., 2016). However, in a total of 56 IVF cycles, a total of only 84 oocytes were obtained, but a fertilization rate of 58% was achieved with embryo transfer in 68% of cycles.

In addition to the potential for natural conception, endogenous hormone production by the graft is also a key benefit of this approach. The great majority of ovarian replacements showed evidence of hormonal activity, although for a variable duration, occasionally up to several years. It is unclear at present what factors predict the duration of the graft, and how much can be related to surgical technique at replacement compared with the intrinsic functional potential of the tissue. Related to this, replacement has also been proposed as a method for inducing puberty (Poirot *et al*., 2012; Ernst *et al*., 2013). This, however, is controversial for a number of reasons (Anderson *et al*., 2013). These include that it results in rapid escalation of oestrogen levels to adult concentrations, in contradiction to the established principles of pubertal induction with slow progression and escalation of oestrogen dose. Additionally, the early onset of ovulatory cycles will expose the patient to progesterone earlier than would occur during normal puberty, which may impact on normal breast development. The tissue also contains a finite number of oocytes, thus this could be argued to be a wasteful use of this scarce resource. There is also the potential risk of malignant contamination, discussed more fully below. However, there is also clear value to women from endogenous and physiological hormone production. The adverse health effects of POI are increasingly recognized and whilst it is very likely that many of these are reduced by appropriate exogenous hormone replacement, there is a paucity of robust evidence regarding the risks and benefits of HRT in women with POI (Langrish *et al*., 2009; Crofton *et al*., 2010), with much of it being extrapolated from women taking HRT after a physiological menopause (ESHRE Guideline Group on POI *et al*., 2016). Replacement of cryopreserved ovarian tissue purely for hormone production rather than fertility has been proposed (Andersen and Kristensen, 2015). This may well be appropriate for some women who have completed their family or no longer wish to pursue fertility, taking into account the risk of malignant contamination, the uncertain duration of function of replaced ovarian tissue, and issues surrounding risks and benefits of hormone ‘replacement’ beyond the age of natural menopause.

## The risk of malignant contamination

**Figure 2:**
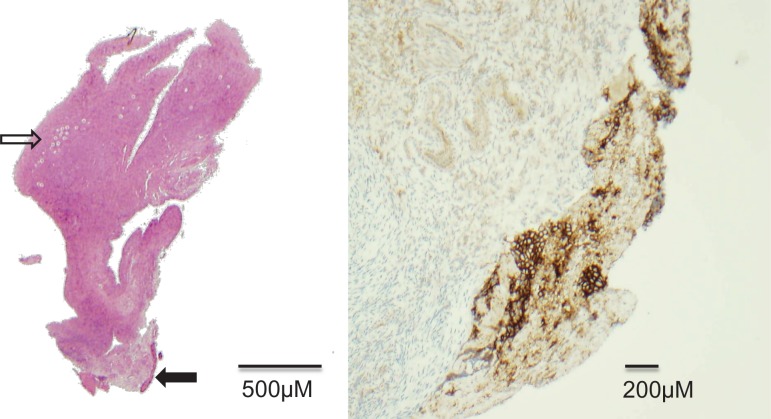
Biopsy of ovary from a girl with Ewing's sarcoma in a vertebra. A 12-year-old with Ewing's sarcoma localized to the T7 vertebra and no evidence of metastatic disease underwent ovarian biopsy for fertility preservation. A biopsy sent for pathological examination showed presumed malignant contamination, with CD99 positive cells present consistent with metastatic Ewing's sarcoma. Left panel, solid arrow indicates area of CD99 positive cells in a haematoxylin and eosin stained section, open arrow indicates primordial follicles in cortex of ovary. Right panel: CD99 (brown stain) immunohistochemistry result.

The storage of ovarian tissue before cancer treatment is started carries with it the risk that the tissue will contain malignant cells, which will survive cryopreservation and could potentially be transplanted back into the patient with the risk of recurrence of the malignancy. A range of methods specific to the diagnosis can be used to detect malignant contamination, including immunohistochemistry and molecular analysis, and a sample of tissue should be sent for such analysis at the time it is stored. These tests are all, however, destructive, and therefore cannot be applied to the actual pieces of tissue that will be replaced in the patient. In general, it is considered that where there is no evidence of metastatic disease of solid cancers, then the risk of ovarian contamination is low. However, for leukaemias before treatment has started there is a high risk that circulating malignant cells will be present in the ovary, and this has been confirmed in a number of studies specifically investigating this (Greve *et al*., 2012; Dolmans *et al*., 2013; Rosendahl *et al*., 2013). This also confirms the potential of these tests to identify malignant contamination although there may be issues of sensitivity to pick up minimal residual disease, which will be expected to improve with time. A high level of vigilance is required to detect contamination even when the diagnosis and staging tests indicate a low risk. In our own series of patients, we have identified a case of ovarian contamination with Ewing's sarcoma that had arisen in a thoracic (T7) vertebra and conventional staging showed no evidence of metastatic disease (Fig. 2). The identification of this contamination will, of course, preclude replacement of the tissue and alternative approaches to the restoration of fertility in such patients are required. These are discussed below. The application of ovarian tissue cryopreservation in patients with leukaemia remains controversial because of this risk of contamination. It has been proposed that ovarian tissue can be taken following initial courses of chemotherapy, potentially sufficient to reduce the risk of cryopreservation of circulating malignant cells but without excessively compromising ovarian function (Greve *et al*., 2012). Whilst successful cases have been reported (Meirow *et al*., 2016), the benefits and risks of this approach have yet to be clearly quantified and undertaking ovarian tissue cryopreservation in these patients remains contentious.

## Developing methodologies to optimize the potential of cryopreserved tissue

Whilst reimplantation of ovarian tissue has been a major advance for fertility preservation, it is not suitable for all patients as there may be concerns about transplanting malignant cells into disease-free patients. With these concerns in mind, a great deal of research has focused on first, isolating follicles from tissue at specific stages of development either before or after cryopreservation, removing any contaminating cells and recombining the follicles within a supportive matrix to form an ‘artificial ovary’ for subsequent reimplantation or *in vitro* development, and second, developing oocytes from cryopreserved tissue completely *in vitro*.

## Making an artificial ovary

The aim of developing an ‘artificial ovary’ is to produce a structure that would be free of any potential malignant cells and contain healthy immature follicles (and stromal cells). Groups working in this area have focused on isolation methods and in developing matrices to support isolated follicles (Skory *et al*., 2015; Paulini *et al*., 2016). Developing the optimal matrix to support follicle/oocyte development has been slow but some advances have been made. The optimal matrix needs to support growth, development and recovery of human ovarian follicles as well as control rigidity. Bioengineering has led to the development of many materials to support follicle development (Shea *et al*., 2014). Alginate has given positive results with mouse and human follicles (Yin *et al*., 2016) and fibrin has also been demonstrated to be a viable material to support isolated murine primordial follicles after transplantation (Kniazeva *et al*., 2015). Decellularized ovary has been used to support the growth and development of mouse follicles (Laronda *et al*., 2015). Recent work with isolated preantral human follicles has demonstrated that a matrix formed by combining fibrin with fibrinogen and thrombin improves the health and development of the encapsulated follicles (Paulini *et al*., 2016). Work in this area is promising but there is still much to be tested before an ‘artificial’ ovary could reach the clinic.

The avoidance of a transplantation procedure would be preferred for some patients. To obtain fully grown competent oocytes from cryopreserved tissue without a transplantation step would require the immature oocytes to be grown completely *ex vivo*.

## Oocyte growth and development *in vitro* using cryopreserved tissue

Growth *in vitro* from the most immature oocytes (primordial stages) with subsequent IVF and production of live young has only been achieved in mouse (Eppig and O'Brien, 1996; O'Brien *et al*., 2003). The complete process of murine oogenesis from the primordial germ cell stage to the production of developmentally competent oocytes can be recapitulated *in vitro* (Morohaku *et al*., 2016) and the resultant offspring have been shown to be healthy. Whilst there are major differences between human and mouse oocyte development, the mouse work provides proof of concept that complete oocyte development can be achieved *in vitro* and has driven the development of culture systems that could be applied to other species, particularly human. Advances in culturing follicles from humans, non-human primates and domestic species have been made, bringing the prospect of achieving an *in vitro* system that supports complete human oocyte development closer (Smitz *et al*., 2010; Telfer and Zelinski, 2013; Xiao *et al*., 2015).

Several culture systems have been developed that have been optimized for a range of different animal models (Smitz *et al*., 2010; Telfer and McLaughlin, 2012; Telfer and Zelinski, 2013). Whilst some are more advanced than others, there is not a single system that has been fully optimized to support the complete *in vitro* growth and maturation of human follicles and oocytes. All of the published systems have strengths and weaknesses that may be usefully exploited to meet the challenges of human follicle culture but there is agreement that to achieve complete *in vitro* growth, a dynamic culture system is required to support the three main transition steps in oocyte/follicle development, i.e. growth activation, ongoing culture of isolated follicles and final oocyte maturation.

## *In vitro* activation of primordial follicles

The regulation of follicle activation involves a combination of inhibitory, stimulatory and maintenance factors (Nelson *et al*., 2013; Hsueh *et al*., 2015). Work using knockout mouse models has highlighted the importance of the phosphatidylinositol-3′-kinase (PI3K-AKT) signalling pathway within the oocyte in regulating follicle activation (Reddy *et al*., 2008). Using pharmacological inhibitors of phosphatase and tensin homologue *in vitro* leads to increased activation of human primordial follicles (Li *et al*., 2010; McLaughlin *et al*., 2014), whereas treatment with rapamycin (an inhibitor of mammalian target of rapamycin complex 1: mTORC) results in decreased activation (McLaughlin *et al*., 2011). A human live birth has been reported following the treatment of ovarian tissue for 48 h *in vitro* with bpV(HOpic) and 740YP, an Akt stimulant, followed by replacement and IVF (Kawamura *et al*., 2013). This is an extremely encouraging development but needs to be treated with caution as similar inhibitors *in vitro*, whilst increasing activation of primordial follicles, result in poor quality oocytes at later stages (McLaughlin *et al*., 2014).

## Isolation and culture of growing follicles to achieve oocyte growth and development

Once follicles have reached the multi-laminar preantral stage of development, there are several culture systems that support further growth. Placing preantral follicles in v-shaped micro-well plates allows maintenance of three-dimensional follicular architecture *in vitro* whilst promoting growth and differentiation of human follicles (Telfer *et al*., 2008; Anderson *et al*., 2014; McLaughlin *et al*., 2014) with antral formation occurring within 10 days. Follicle encapsulation in alginate hydrogels has also been used to support secondary human (Xu *et al*., 2009; Xiao *et al*., 2015) and rhesus monkey (Ting *et al*., 2011) follicle growth *in vitro*. Alginate encapsulation is believed to mimic the extracellular matrix *in vivo* in terms of its ability to facilitate molecular exchange between the follicle and the culture medium, and whilst its flexibility can accommodate cell proliferation, its rigidity prevents dissociation of the follicular unit. The progression of human follicles following isolation from the cortex is remarkable. In the presence of FSH, enzymatically isolated secondary human follicles can differentiate, become steroidogenically active and complete oocyte growth in 30 days (Xu *et al*., 2009) and have been shown to be capable of meiotic maturation (Xiao *et al*., 2015).

## Aspiration and maturation of oocyte cumulus complexes

The final goal of an *in vitro* system is to produce oocytes that can be fertilized and produce developmentally normal embryos. In order to achieve this, *in vitro* grown human oocytes need to be meiotically matured *in vitro* and several groups have made important steps towards this for clinical use over the last two decades (Alak *et al*., 1998; Cha and Chian, 1998; Mikkelsen *et al*., 1999; Cavilla *et al*., 2008; Chian *et al*., 2013). It is widely accepted that whilst 40–80% of immature human oocytes can successfully complete IVM and fertilization, the rate of maturation of immature oocytes is still well below that of oocytes harvested from stimulated ovaries, indicating that the protocols are suboptimal or many of the harvested oocytes are intrinsically unable to undergo maturation (Chang *et al*., 2014).

**Figure 3:**
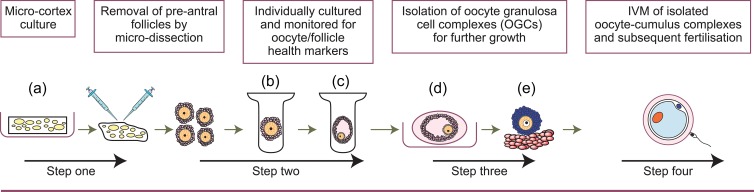
Multistep culture system for activation of human primordial follicles and subsequent follicle/oocyte development. Primordial follicles can be activated or held dormant within ovarian cortical tissue (**a**). Once activated preantral stages can be isolated (**b**), they are grown individually to antral stages (**c**). These can be further grown, for example, within an alginate bead (**d**) to obtain fully grown oocytes (**e**), then competence to undergo maturation and fertilization is tested.

The challenge in developing a complete *in vitro* growth system is to combine each of these stages to produce a multistep system that supports the complete process of oocyte development (Fig. 3).

## Conclusion

Preservation of fertility in girls and young women with cancer who are at risk of loss of fertility remains an important priority to improve their long-term health and well-being. Oocyte and embryo cryopreservation are established but in most countries in the world, ovarian tissue cryopreservation remains experimental, particularly in younger adolescents and prepubertal girls. Cryopreservation of ovarian cortical tissue with later replacement has now resulted in at least 100 live births but requires an additional invasive procedure at a time of emotional distress and uncertainty, and there is an uncertain risk of tissue contamination in haematological and other malignancies. *In vitro* growth and maturation of immature ovarian follicles is developing but further research is required before it can be used for clinical benefit. Girls and young women having ovarian tissue cryopreservation should be counselled and have their data collected in a research context, to improve the evidence base underpinning this approach. This field requires more basic and clinical research to provide accurate information for patients and their medical teams and to promote the development of ovarian cryopreservation as an effective and cost-efficient treatment option for female fertility preservation.
